# Mesoscopic Modeling and Experimental Validation of Thermal and Mechanical Properties of Polypropylene Nanocomposites Reinforced By Graphene-Based Fillers

**DOI:** 10.1021/acs.macromol.3c01529

**Published:** 2023-12-06

**Authors:** Atta Muhammad, Rajat Srivastava, Nikolaos Koutroumanis, Dionisis Semitekolos, Eliodoro Chiavazzo, Panagiotis-Nektarios Pappas, Costas Galiotis, Pietro Asinari, Costas A. Charitidis, Matteo Fasano

**Affiliations:** † Department of Energy, Politecnico di Torino, Corso Duca degli Abruzzi 24, 10129, Torino, Italy; ‡ Department of Mechanical Engineering, Mehran University of Engineering and Technology, SZAB Campus, 66020 Khairpur Mir’s, Sindh, Pakistan; § Department of Engineering for Innovation, University of Salento, Piazza Tancredi 7, 73100, Lecce, Italy; ∥ Foundation of Research and Technology-Hellas, Institute of Chemical Engineering Sciences, Stadioustr Rion26504, Patras, Greece; ⊥ School of Chemical Engineering, National Technical University of Athens, 9 Heroon Polytechniou, 15780 Athens, Greece; # Department of Chemical Engineering, University of Patras, 1 Caratheodory26504 Patras, Greece; ∇ Istituto Nazionale di Ricerca Metrologica, Strada delle Cacce 91, 10135 Torino, Italy

## Abstract

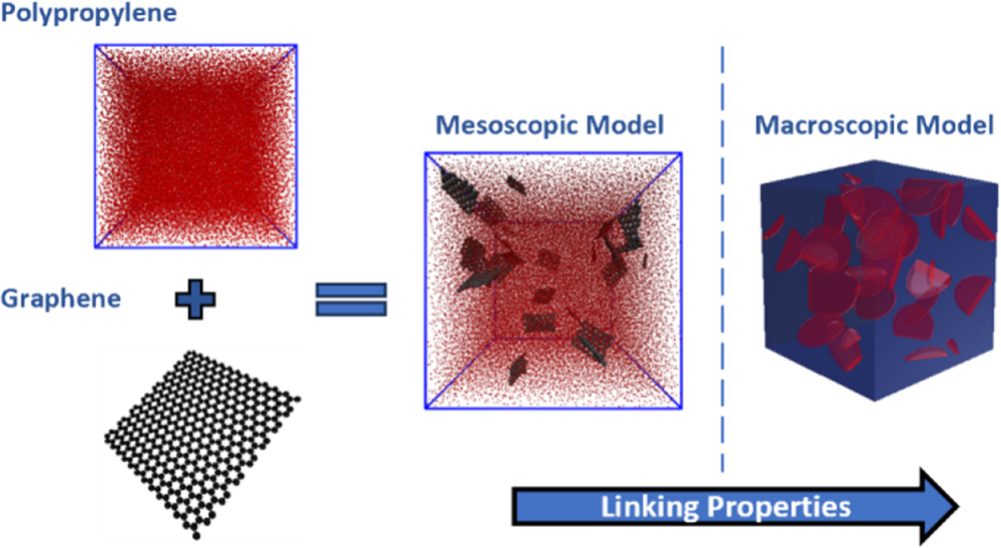

The development of nanocomposites relies on structure–property relations, which necessitate multiscale modeling approaches. This study presents a modeling framework that exploits mesoscopic models to predict the thermal and mechanical properties of nanocomposites starting from their molecular structure. In detail, mesoscopic models of polypropylene (PP)- and graphene-based nanofillers (graphene (Gr), graphene oxide (GO), and reduced graphene oxide (rGO)) are considered. The newly developed mesoscopic model for the PP/Gr nanocomposite provides mechanistic information on the thermal and mechanical properties at the filler–matrix interface, which can then be exploited to enhance the prediction accuracy of traditional continuum simulations by calibrating the thermal and mechanical properties of the filler–matrix interface. Once validated through a dedicated experimental campaign, this multiscale model demonstrates that with the modest addition of nanofillers (up to 2 wt %), the Young’s modulus and thermal conductivity show up to 35 and 25% enhancement, respectively, whereas the Poisson’s ratio slightly decreases. Among the different combinations tested, the PP/Gr nanocomposite shows the best mechanical properties, whereas PP/rGO demonstrates the best thermal conductivity. This validated mesoscopic model can contribute to the development of smart materials with enhanced mechanical and thermal properties based on polypropylene, especially for mechanical, energy storage, and sensing applications.

## 1 Introduction

In recent decades, polymers have been used in several industrial applications, ranging from the medical to the automotive industry, because of their light weight, corrosion resistance, low cost, and ease of manufacture.^1,2^ Thermoplastics account for roughly 76% of the polymers used globally.^3^ The overall consumption of polypropylene (PP) is low compared to that of other thermoplastics such as polyethylene; however, its consumption has increased significantly in recent years owing to its interesting physical and chemical properties.^3^ Polypropylene, an olefin, is partially nonpolar and crystalline. It is fabricated from the propylene monomer by chain-growth polymerization. The chemical formula for polypropylene is (C_3_H_6_)_*n*_, and it is currently one of the low-priced polymers.^4^ It can be processed through extrusion and injection molding.^4,5^ Generally, polypropylene has a relatively low mechanical strength and poor thermal conductivity compared to high-performance polymers.^6,7^ However, typically, it shows better processability, which makes it easier to melt, shape, and mold. This can lead to faster production and reduced manufacturing complexities. Not all applications require the performance of high-value polymers, and polypropylene-based composites might be sufficient for certain applications where heat resistance or chemical resistance is not a critical factor. Using a lower-cost polymer with the fillers can strike a better balance between performance and affordability for certain applications such as aerospace,^8^ automotive,^9^ food packaging,^10^ medical devices,^11^ and energy applications.^12^

Nanofillers can be included into the polypropylene matrix to form a composite with enhanced thermal and mechanical properties.^13,14^ Polypropylene matrix can be reinforced with different nanofillers, such as glass fibers, aluminum oxide nanoparticles, and carbon nanotubes,^15−17^ to achieve desirable thermal and/or mechanical properties. For instance, Mirjalili et al.^16^ performed morphological and mechanical characterization of polypropylene/nano α-Al_2_O_3_ composites. They observed an increase in the elastic constant of PP by increasing the nano α-Al_2_O_3_ content in the PP matrix from 1 to 4 wt %. Further increase in the loading of nano α-Al_2_O_3_ led to a reduction in the elastic constant because of the agglomeration of α-Al_2_O_3_ nanoparticles. Funck and Kaminsky^18^ studied the multiwalled carbon nanotube (MWCNT)-reinforced polypropylene nanocomposites by in situ polymerization. Different MWCNT concentrations (0.1–8.0 wt %) were introduced into the polypropylene matrix. They investigated their characteristics, such as morphology, crystallization and melting temperatures, and half-time of crystallization, and found that the half-time of crystallization decreases significantly as the filler content increases.

Given their superior properties, if introduced into the PP matrix, graphene-based nanofillers (e.g., graphene, graphene oxide, and reduced graphene oxide) can significantly improve the material characteristics (such as elastic, thermal, and electrical properties) even at small concentrations.^19−22^ Graphene (Gr) is a carbon allotrope comprising covalently linked carbon atoms bonded via sp^2^ orbitals and structured in a two-dimensional hexagonal lattice.^23^ Graphene oxide (GO) is a graphene derivative that has variable ratios of oxygen-rich functional groups on the basal plane and free edge, such as epoxide, carbonyl, carboxyl, and hydroxyl groups.^19^ When compared to pristine graphene, the presence of the functional group in GO weakens its in-plane mechanical properties, such as elastic constant and intrinsic strength. GOs have been used in polymer nanocomposites because the thermal and electrical properties of polymers can be significantly enhanced by the incorporation of GO nanosheets.^24^ Owing to its cost-effectiveness and ease of production, reduced graphene oxide (rGO) is also commonly used as a filler with various materials to prepare nanocomposites. rGO is obtained from the reduction of graphene oxide using chemical, thermal, or photothermal reduction methods.^24^ The fraction of the oxidized group is lowered when graphene oxide is reduced to obtain rGO, resulting in structural defects.^24^ Nanocomposites with GO and rGO can be used for energy storage, stimuli-responsive materials, anticorrosion coatings, and separation applications.^24^

The technological development of nanocomposites with desired properties strongly depends on a deep understanding of the structure–process–property relationship with molecular precision.^25^ Computer modeling is emerging as a powerful supplement to experimental and analytical approaches, which can provide improved understanding of the multiscale behavior of complex materials.^26,27^ Multiscale modeling strategies provide seamless coupling among various lengths and time scales of material properties and structures, from atomistic to mesoscopic and then to continuum scale.^28^ At the atomistic level, molecular dynamics (MD) simulations have been utilized to investigate the behavior of the nanocomposite constituent elements (such as polymer matrices and nanofillers) and their interaction at the interfaces.^29,30^ The properties of the constituents of nanocomposites obtained at the atomistic level can then be employed in the mesoscopic model. The mesoscopic structure of a nanocomposite is represented by the representative volume element (RVE) of the material, and the properties computed at the mesoscale can be finally homogenized to evaluate the effective thermal and mechanical properties at the macroscopic (continuum) level.^29^

Molecular dynamics simulations have allowed for the anticipation of material properties with well-parametrized and validated interatomic potential. Polymers have also been investigated using MD simulations with an accurate computation of their thermal and mechanical properties.^31−33^ Wang et al.^34^ performed MD simulation to observe the effects of molecular weight, chain number, and cooling rate on the glass transition temperature and coefficient of thermal expansion of poly(ethylene oxide). They found that the density increases with the chain length and thus molecular weight. Also, the glass transition temperature increased as the cooling rate increased, consistent with the experimental evidence.^35,36^ Kim et al.^37^ determined the hygroscopic and mechanical properties of semicrystalline polypropylene using molecular dynamics and compared the results with experimental data. In particular, the elastic modulus and moisture saturation were studied with respect to the degree of crystallinity, and they concluded that the elastic modulus obtained from the MD simulation shows a trend similar to the experimental results and increases with degree of crystallinity. In the case of moisture absorption, it was determined that a higher degree of crystallinity caused lower moisture uptake from both the MD simulation results and the experiments. Guryel et al.^38^ used MD simulation to study the morphological and structural properties of three different polymeric nanocomposites reinforced with graphene. The polymers used in their study are polyethylene (PE), polystyrene (PS), and polyvinylidene fluoride (PVDF). They found the PE to have higher crystallinity than PVDF at a temperature of 500 K. Graphene influenced the crystallization of PVDF and PE because it acts as a nucleation site in both polymers. Their results were in line with those obtained by a previous quantum mechanical study.^39^ Zhang et al.^40^ also performed an MD simulation of glass fiber-reinforced polypropylene composites under various dynamic and thermal loadings. The interfacial strength decreases as the temperature increases, resulting in a reduction in the mechanical properties of the matrix, whereas the mechanical properties increase with the strain rate.

Generally, atomistic models are computationally expensive and are limited to certain lengths and time scales. A possible solution to these problems is adopting mesoscopic coarse-grained (CG) models, which cluster groups of homogeneous atoms into one bead, thus reducing the degrees of freedom of the system.^31,41^ In recent years, the MARTINI coarse-grained model^42,43^ has been an effective model for simulating polymers including polypropylene. In the MARTINI model, four heavy atoms and their accompanying hydrogens are represented by a single interaction center on average.^44^ Panizon et al.^45^ developed a CG model for polypropylene using structural and thermodynamic characteristics as an earmark in the parametrization. As goal parameters, they considered densities and the radii of gyration for structural properties and segmentation of the various building blocks for thermodynamic properties, and the model was validated by matching the structural characteristics of the polymer. Ruiz et al.^46^ developed a CG model of graphene based on the strain energy conservation technique, where the model potentials are adjusted using the mechanical properties of graphene. The model can simulate mechanical responses in both the elastic and fracture domains. They found that the present model can be used for graphene-based nanocomposites. Similarly, Meng et al.^19^ presented a CG model of graphene oxide using the strain energy conservation approach to optimize the potential parameters based on DFT calculations. They identified that the model could capture the mechanical and interfacial properties as well as the effect of oxidation in GO sheets and hence is appropriate for inspecting the mechanical and interfacial properties of GO-based nanocomposites.

However, to the best of the authors’ knowledge, the properties of PP nanocomposites reinforced by graphene fillers have never been investigated by CG-MD. The present study proposes a new mesoscopic approach to determine the thermal and mechanical properties of polypropylene nanocomposites reinforced by graphene-based nanofillers. Initially, we determined the thermal and mechanical properties of pure polypropylene- and graphene-based nanofillers (Gr, GO, and rGO). Then, the influence of the graphene-based nanofiller reinforcements on the thermal and mechanical properties of polypropylene was assessed. To compare the accuracy of the CG-MD model of PP/Gr nanocomposites with respect to traditional continuum approaches, finite element and mean field simulations were also carried out. Finally, an experimental thermomechanical characterization of polypropylene–graphene nanocomposites was conducted to validate the proposed multiscale modeling approach, where the peculiar properties of the filler–matrix interface quantified by the mesoscopic model are employed to enhance the accuracy of continuum models.

## 2 Methods

### 2.1 Mesoscopic Models

**Figure 1:**
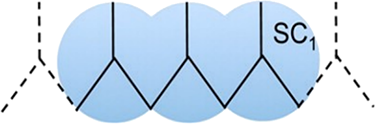
Coarse-grained representation of polypropylene (blue beads) from atomic details (black lines), where SC_1_ represents standard MARTINI beads. Reproduced from ref (45) with permission from ACS Publications.

The considered coarse-grained model of polypropylene is taken from Panizon et al.,^45^ who studied the interaction between PP and lipid membranes. They employed 3:1 mapping, as CH_2_ groups are shared by neighboring CG beads as shown in Figure 1. The model contains bond, angle, and dihedral interactions. Harmonic functions describe the bonds and angles, whereas the sum of two proper dihedral functions describes the PP dihedrals, namely,
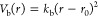
1
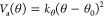
2
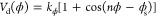
3

In eq 1, *r*_0_ represents the equilibrium distance between the bonded beads, whereas *k*_b_ represents the harmonic constant of their bond. In eq 2, θ_0_ is the equilibrium angle between a triplet of bonded beads, and *k*_θ_ is the angular harmonic constant. In eq 3, *k*_ϕ_, *n,* and ϕ_s_ are the parameters of the proper dihedral potential. The nonbonded interactions of PP are defined by the 12–6 Lennard–Jones potential with a cutoff value of 1.5 nm:
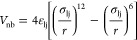
4with ε_lj_ and *r*_lj_ being the energy well and equilibrium distance of the 12–6 Lennard–Jones potential between two nonbonded beads, respectively. The considered parameters of the CG force-field of PP are listed in Tables S1 and S2.

**Figure 2:**
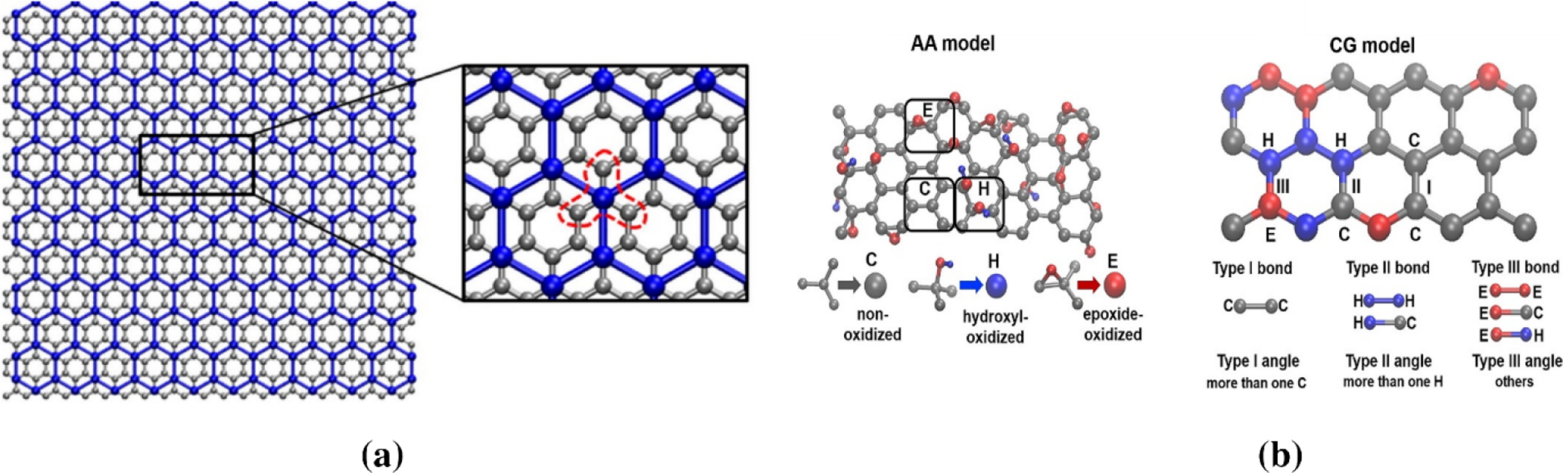
(a) Coarse-grained model for graphene; reprinted from ref (46) with permission from Elsevier. (b) Coarse-grained model of graphene oxide; reprinted from ref (19) with permission from Elsevier.

The CG model of graphene, instead, is provided by Ruiz et al.^46^ It follows the approach of MARTINI, where four atoms are clustered into a single bead as shown in Figure 2a and preserves the hexagonal lattice of the beads.^47^ The CG force field of the graphene model includes bonded and nonbonded interactions. Bonded interactions comprise bonds, angles, and dihedrals. On the one side, the bonding potential is now considered as
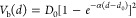
5where *D*_0_ and α parameters are related to the depth and width of the potential well of the bond, respectively, and *d*_0_ represents the equilibrium distance of the bond. On the other side, eqs 2 and 3 are adopted to model angle and dihedral interactions, respectively. Nonbonded interactions are modeled by eq 4 as well. The considered parameters of the CG force-field of graphene are listed in Tables S3 and S4.

Concerning GO, the CG model is inspired from Meng et al.,^19^ who developed it for studying the mechanical behavior of graphene oxide. Like the MARTINI approach, a 4:1 mapping scheme has been followed in GO, keeping the hexagonal structure of pure graphene also in this case. The CG model of GO includes the hydroxyl- and epoxide-oxidized functional groups as well as the nonoxidized group as shown in Figure 2b. The key characteristic that governs the mechanical behavior of the GO is the degree of oxidation. The degree of oxidation is defined as the total percentage of both hydroxyl- (type H) and epoxide-oxidized (type E) beads. The force field of the graphene oxide model includes bonds, angles, and nonbonded interactions. Accordingly, three bond and three angle types exist: nonoxidized, hydroxyl-oxidized, and epoxide-oxidized. The considered potential equations and related parameters of the CG force-field of GO are listed in Table S5. For the CG model of rGO, the same functional forms and parameters of GO have been utilized.

Coarse-grained MD simulations were run with a time step of 1 fs. The LAMMPS package^48^ was used to perform the energy minimization and molecular dynamics calculation. The Nosé–Hoover barostat^49^ and thermostat^50^ were used to control the pressure and temperature. The Verlet algorithm^51^ was employed to integrate the equation of motions. VMD^52^ was used to visualize the model and results. Initially, the models were annealed in an NVT ensemble at 500 K for 1 ns. After that, the simulation box was compressed in the NPT ensemble at 5 atm and 300 K for 1 ns and then equilibrated in an NPT ensemble at 1 atm and 300 K for 10 ns. Finally, CG-MD simulations were run to determine the thermal and mechanical properties of the equilibrated models. The developed codes and numerical protocols are fully available at the Zenodo archive associated with this work.^53^

### 2.2 Computation of Material Properties

The glass transition temperature is a significant physical property of polymeric materials. It determines whether the polymeric material exhibits glassy- or rubbery-like behavior as well as the processing and working temperature range of the polymer. In the performed CG-MD simulations, the glass transition temperature is determined from the change in density or specific volume as a function of temperature at constant particle number, pressure, and temperature. This is because density and temperature have distinct linear relationships above and below the glass transition temperature.^54^

Mechanical properties, such as Young’s modulus and Poisson’s ratio, of the modeled materials are also computed in the present study. In the CG-MD simulation, uniaxial deformation is applied to the system, and the mechanical response of the system is recorded. The Young’s modulus of the equilibrated model is calculated from the uniaxial tensile test, whereas the Poisson’s ratio is obtained using the theory of elasticity based on the Young’s modulus.^55^ The deformation processes are carried out in three different directions, *x*, *y*, and *z*, at a temperature of 300 K. Polymers are typically isotropic materials, implying that the material properties are constant in each direction. Similarly, composites remain isotropic when the fillers are added randomly, whereas they may show anisotropic properties when fillers are aligned in a particular direction. Isotropic materials have only two independent variables (elastic constants) in their stiffness and compliance matrices, whereas anisotropic materials may have up to 21 elastic constants. For the isotropic case, the two elastic constants are Young’s modulus, *E*, and the Poisson’s ratio, *v*, which are related as
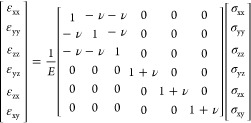
6where σ is the stress vector and ε is the strain vector.

The Müller–Plathe method is used to investigate the thermal conductivity (λ) of CG models. The method entails setting up two cold regions at opposite ends of the simulation box. A certain amount of heat is applied in the central region (hot section), thus inducing a temperature gradient in the model. Velocities exchanged between the atoms of the hot and cold regions generate heat flux. Periodic boundary conditions are applied in all three directions. Once the system reaches steady state, the amount of energy per unit time and cross-sectional area transferred from the hot region to the cold region via velocity exchanges between the molecules is^56^
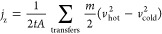
7where *t* is the simulation time, *A* is the cross-sectional area normal to the heat flux direction, *m* is mass, and *v*_hot_ and *v*_cold_ are the velocities of the defined atoms. As a result, *j*_z_ induces a temperature gradient (∇*T*) throughout the system, whose thermal conductivity can be then determined considering Fourier’s law:^56^
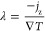
8

Finally, the specific heat capacity of the models at a constant pressure (*c*_p_) is also computed. In the CG-MD simulation, average enthalpies were recorded for every temperature step, and the *c*_p_ of the material was determined from the slope of a linear fit of the resulting enthalpy–temperature plot.

### 2.3 Continuum Models

Two different continuum models, mean field (MF) and finite element method (FE), were used in this study. MF homogenization is based on the first-order Mori–Tanaka (based on an approximation of the Eshelby solution), so it does not require the RVE model and meshing. RVE models were generated for the PP/Gr nanocomposites to perform finite element analysis. In the pursuit of comprehensive continuum modeling that incorporates matrix–filler interactions and thermal boundary resistance, an interphase Mori–Tanaka MF model was adopted. This model casts the interphase as a defined coating enveloping the nanofiller, the thickness of which is precisely set at 0.5 nm, a parameter mechanistically derived from the mesoscopic simulations. Within the domain of the micromechanical model, this interphase plays a pivotal role in enhancing the elastic modulus, thereby imparting a heightened mechanical integrity to the composite material. Conversely, in the thermal model, this interphase assumes the critical function of a thermal boundary resistance, effectively moderating the heat transfer characteristics when juxtaposed with the surrounding host matrix. This duality in the interphase behavior shapes the overall material properties, making it a fundamental element to predict the effective thermal and mechanical properties, of the composite.

In FE, the RVE model is composed of a PP matrix and graphene particulate (platelet-like shape) inclusions with a completely bonded interface and an aspect ratio of 10 (like the CG model). The constitutive behavior of the RVE model replete with isotopically symmetric elements embedded in the nanocomposites adheres to the principles of generalized Hook’s law. Parameters such as the elastic modulus, Poisson’s ratio, and thermal conductivity of these models were computed. Periodic boundary conditions were applied to the RVE models. A tetraconforming mesh incorporating quadratic elements, internal coarsening, and curvature control was used to randomize the distribution of the inclusion phase while maintaining a consistent mass fraction of the CG-MD simulation.

### 2.4 Experimental Methods

We also investigated the experimental thermomechanical characteristics of pure PP and PP samples filled with different graphene concentrations (0.5 and 1.0 wt %) to validate the modeling results. To characterize the mechanical properties, a tensile test was performed. For the sample preparation, isotactic polypropylene was supplied by Sigma-Aldrich, with an average molecular weight Mw = 250,000, number of molecules Mn = 67,000, melting point range 158–170 °C, melt flow index (MFI) 12 g/10 min, and density 0.900 g/cm^3^. The raw material for the nanofillers was graphite powder obtained from NGS Naturgraphit GmbH, with a particle average lateral size of 500 μm. The graphene was then produced using a commercially available shear laboratory mixer by Silverson. The mass of the produced exfoliated graphene was measured after being dried at 80 °C for 24 h under vacuum conditions. The carbon content of produced graphene platelets was approximately 91%, with lateral dimensions in the range of 2–5 μm and a thickness of 5–7 nm (see Figures S1 and S2). PP samples containing 0, 0.5, and 1.0 wt % of graphene were prepared using a hot press. For the preparation of the polymeric film of PP, approximately 10 g of PP at 160 °C was heated using a hot press for about 5 min and then pressed at a high pressure of 50 bar. The procedure was followed 10 times to obtain a homogeneous material. After heating and pressing, the film was taken out of the hot press and then quenched in ice to obtain an amorphous structure and prevent crystallization. In fact, amorphous PP typically shows better processability, faster production, and isotropic properties. A similar procedure, with an additional step of melt (pre)mixing of nanoinclusions into the polymer matrix, has been adopted for the PP/Gr composite with two proportions: 0.5 and 1.0 wt %.

**Figure 3:**
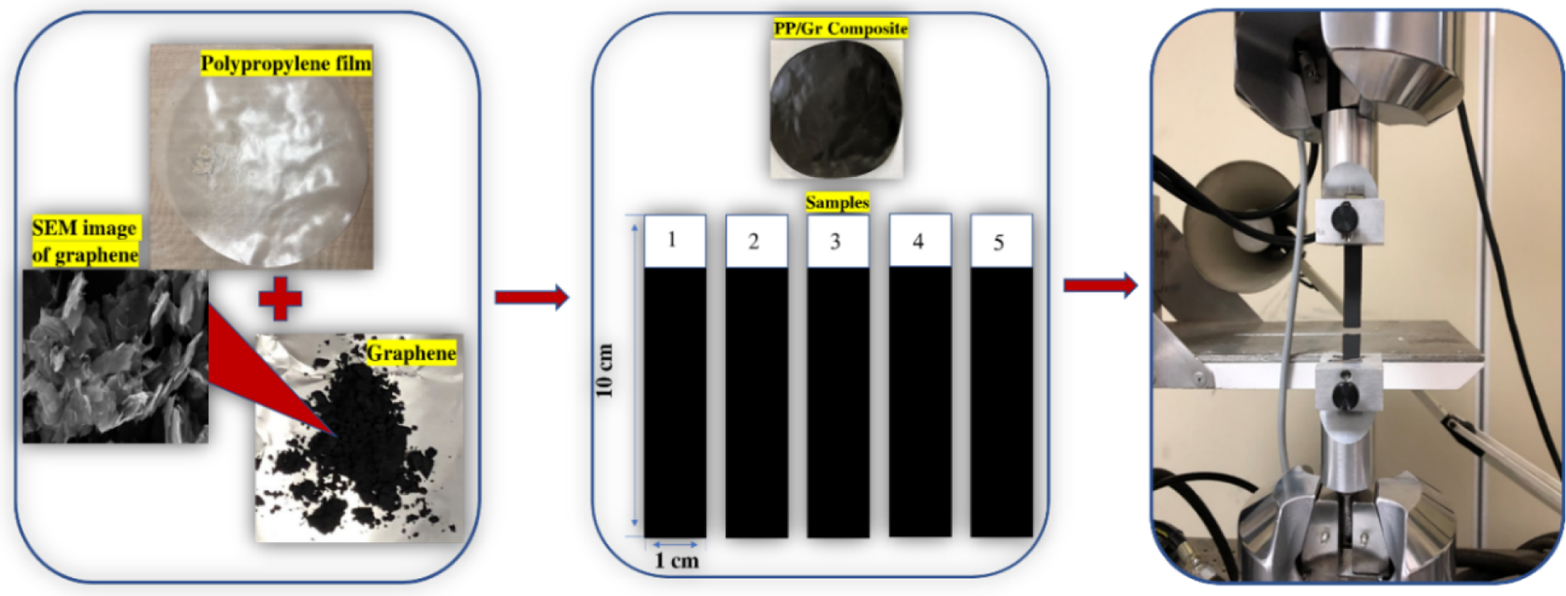
Mechanical characterization of the PP/Gr composite.

Specimens of PP and PP/Gr were prepared following the international standard test method for the tensile properties of polymers (ASTM D882-02). They were machined into 10 × 1 cm^2^ rectangle specimens. The thicknesses of these samples were measured at various lengths, and the average was recorded; the area of each strip was calculated to determine the stress acting on the strips. Specimens were tested by using a mechanical tensile system at 300 K and 1 atm at a strain rate of 25 mm/min. For statistical purposes, five samples of each composition were tested, and the average values of Young’s modulus were obtained, as shown in Figure 3.

For investigating the thermal conductivity of pure PP and PP/Gr (0.5 and 1.0 wt %) composites, test specimens with 30 mm length, 30 mm width, and 5 mm thickness were taken. Two samples of each composition were prepared. The measurements were performed five times for each sample using the transient plane source (TPS) method and the hot disk thermal constant analyzer instrument at ambient *T* (∼25°*C*). Thermal properties of the polymer samples were examined according to the international standard ISO 22007 for the TPS method and the hot disk thermal constants analyzer instrument.^57^ The sensors are positioned between the plane surfaces of two sample pieces of the material being studied. The hot disk sensor is made of a double spiral electrically conductive pattern etched from a thin sheet of nickel. The basic principle of the system is to constantly supply power to an initially isothermal sample via a hot disk sensor and then use the same sensor as a resistance thermometer to follow the consequent temperature rise throughout a specified heating period. The dynamic characteristics of the temperature rise, reflected in sensor resistance increments, were carefully recorded and analyzed, allowing for the determination of both the thermal conductivity and thermal diffusivity from a single transient recording.

## 3 Results and Discussion

### 3.1 Polypropylene

**Figure 4:**
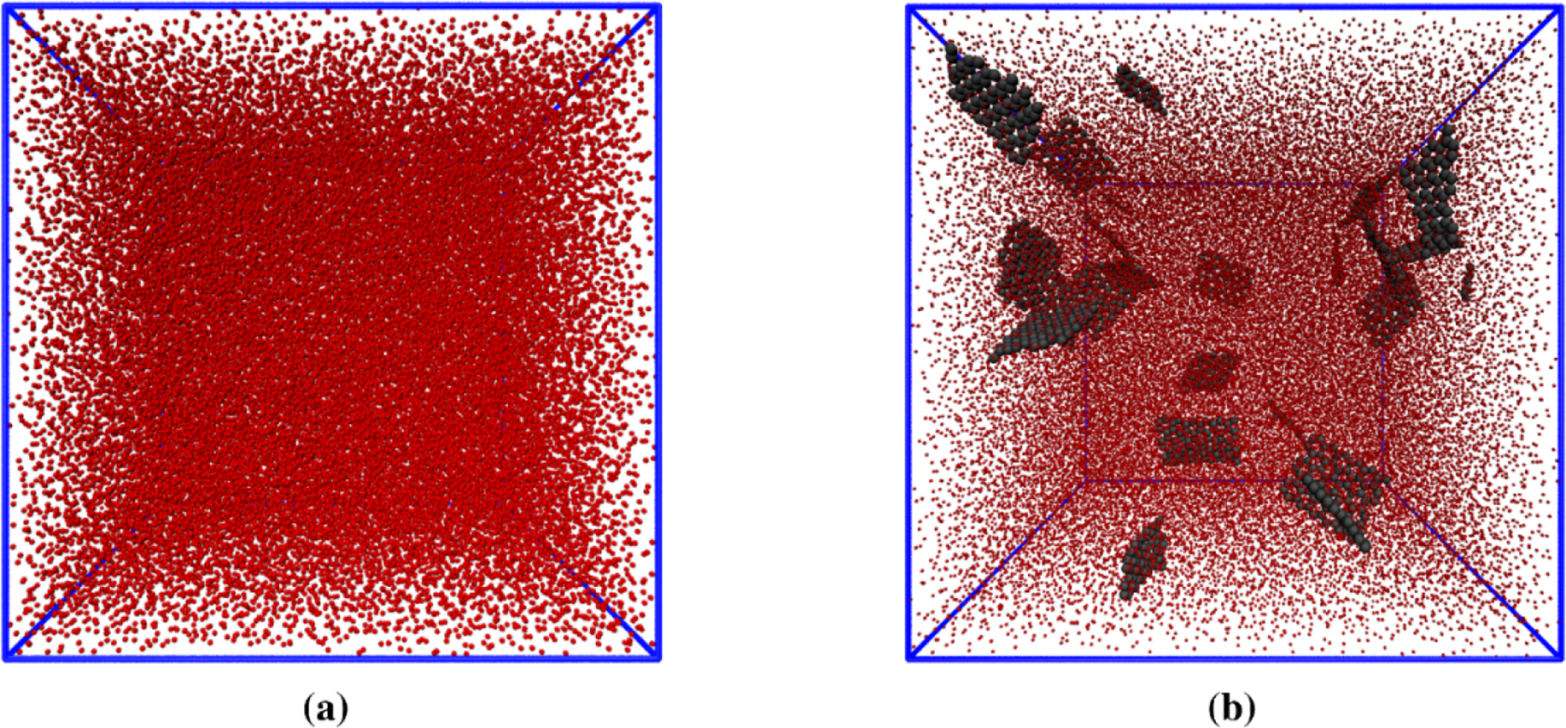
Equilibrated CG model for (a) pure PP and (b) PP/Gr nanocomposite (2.0 wt % Gr).

**Figure 5:**
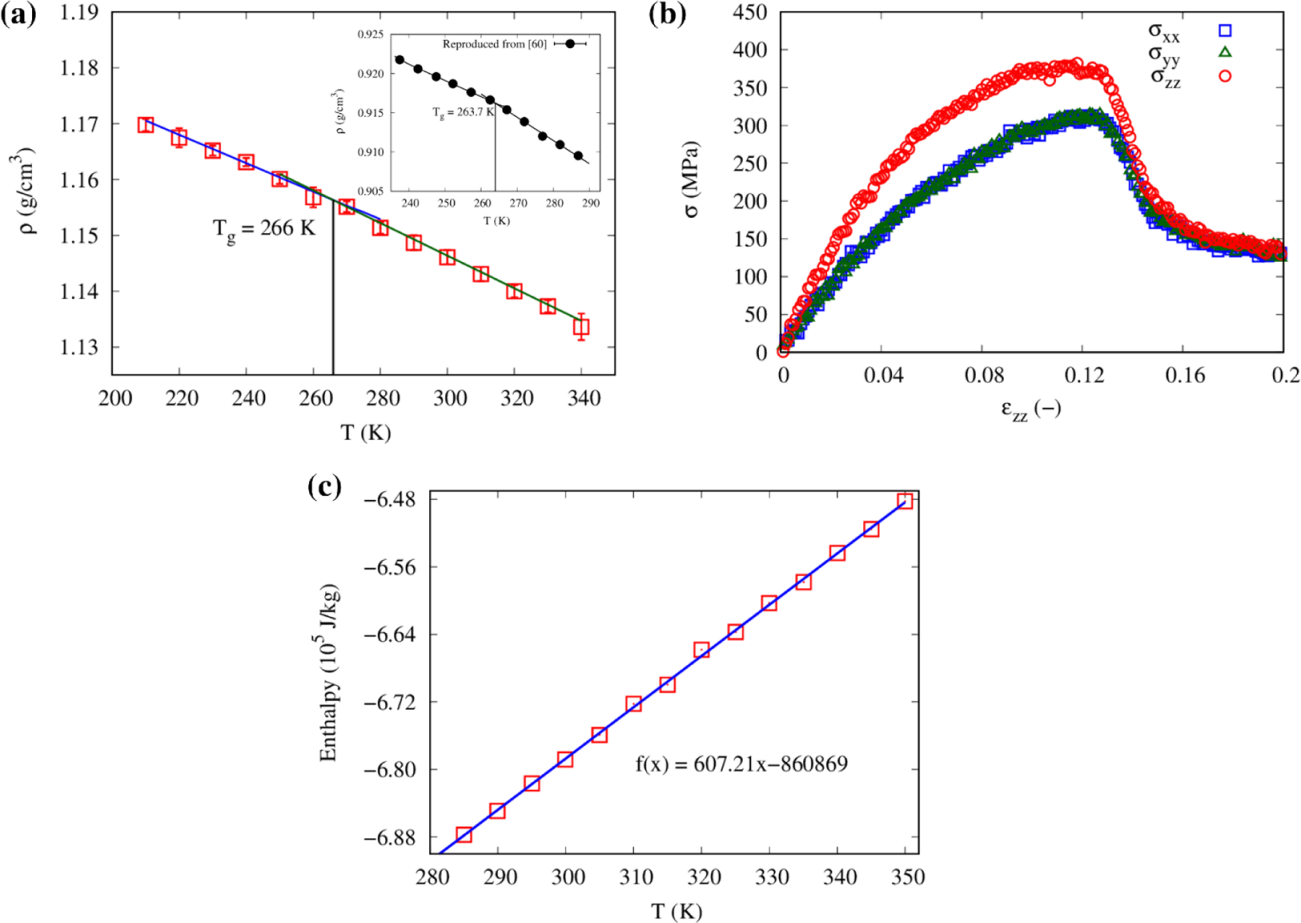
(a) Density as a function of temperature (error bars represent ±1 SD), (b) stress vs strain curve, and (c) enthalpy–temperature plot for the CG model of pristine polypropylene. The inset of panel a reports the experimental data points from ref (60) .

Boxes of 400 PP chains, with each chain containing 150 repetitive CG beads, as shown in Figure 4a, are equilibrated by performing NVT and NPT ensemble simulations for 10 ns. The density of PP computed at different temperatures (210–350 K) was found to be in the range of 1.13–1.17 g/cm^3^, which is higher than the values reported in literature (0.90–0.91 g/cm^3^).^58^ This overestimation of density using MARTINI force field is also reported in previous studies.^45^ In fact, the hydrophobic nature of PP suggests using the most hydrophobic MARTINI beads (C_1_); however, because PP beads are connected by a short bond length (0.29 nm), the smaller SC_1_ beads are considered to represent each monomer. This choice leads to poor performance (overestimation) in terms of density.^45^ Using densities at varied temperature levels obtained from the NPT ensemble at a pressure of 1 atm, the glass transition was calculated by determining the intersection point between two fitting lines against the density–temperature plot, as shown in Figure 5a. The simulated glass transition temperature of PP (*T*_g_ = 261–266 K) is in good agreement with data from previous studies (*T*_g_ = 259–263 K).^59,60^

Mechanical properties such as Young’s modulus and Poisson’s ratio are then extracted from stress–strain curves. The stress–strain curves obtained from the uniaxial tensile deformation in the *x*, *y*, and *z* directions are shown in Figure 5b. The value of Young’s modulus (0.99 GPa) is in line with our experimental result (0.94 GPa, see Figure S3) and literature value (1.05 GPa).^61^ Similarly, the computed Poisson’s ratio (0.43) is in good agreement with the literature value (0.42).^62^ The specific heat capacity of the PP was also computed from the enthalpy–temperature plot, as shown in Figure 5c. The best linear fit of this plot had a slope of 607 J/(kg K), whereas the experimentally measured specific heat capacity of PP was 1,700 J/(kg K) at a temperature of 300 K.^63^ We also determined the thermal conductivity of neat polypropylene in all three directions. Results show that the average thermal conductivity of polypropylene at 300 K is 0.13 W/(m K), which is lower than our experimentally measured value (0.23 W/(m K)) but in the range reported in the literature (0.11–0.22 W/(m K)).^64,65^

### 3.2 Graphene-Based Fillers

**Figure 6:**
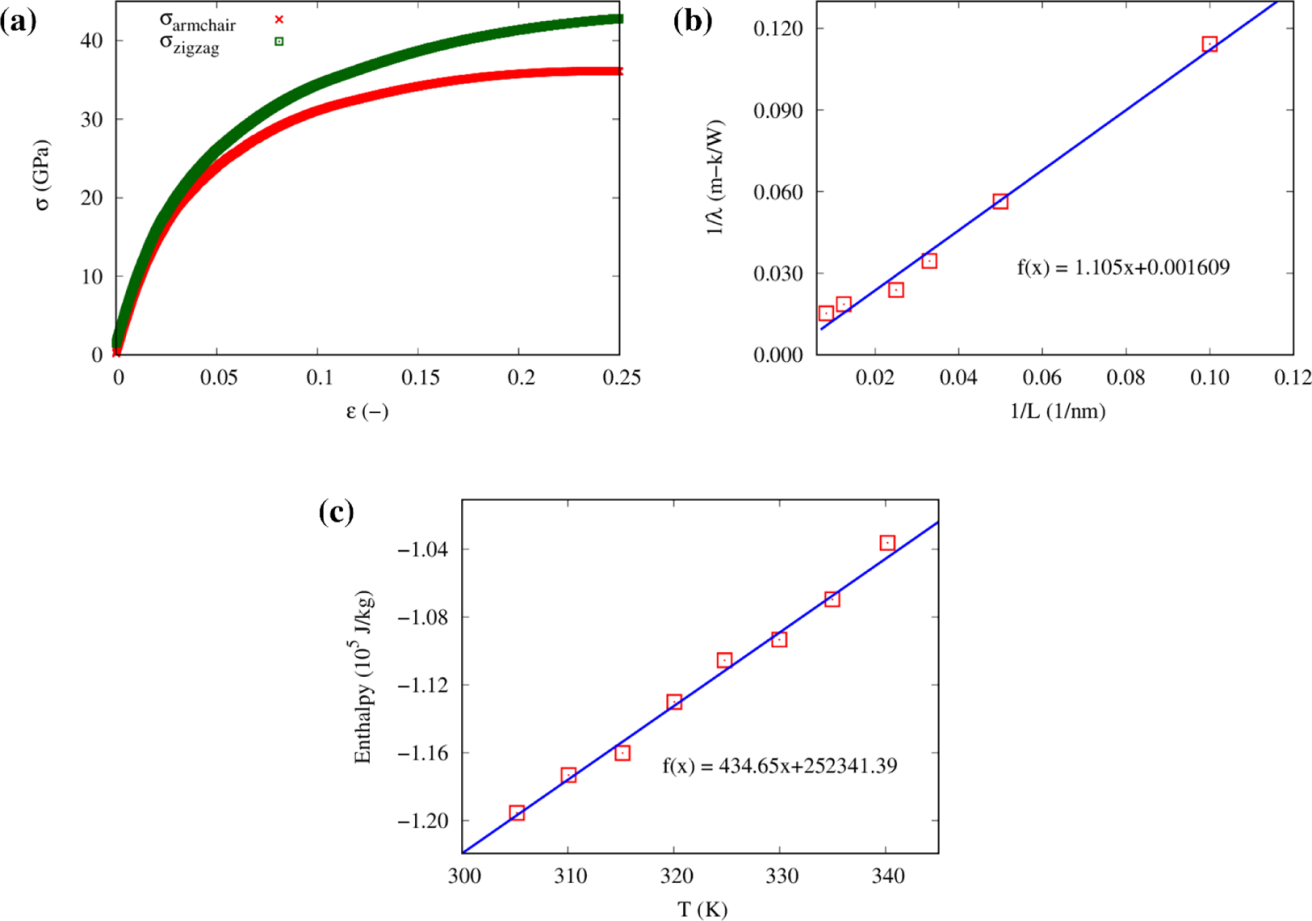
(a) Stress vs strain response of single CG graphene sheet in armchair and zigzag directions, (b) inverse of thermal conductivity–inverse of length curve in armchair direction, and (c) enthalpy–temperature plot.

CG-MD simulations are then used to compute the thermal and mechanical properties of single-layered graphene sheets in armchair and zigzag directions. The initial configuration of CG graphene was generated by VMD considering the bond length of the CG model of graphene. The considered size of the graphene sheet was 20 × 40 nm^2^. The *x-* and *y*-axis directions correspond to the zigzag and armchair edges, respectively. Initially, the system was equilibrated using an NVT ensemble for 100 ps. After the equilibration, uniaxial tensile deformations along the armchair and zigzag directions were applied to the system, as shown in Figure 6a.

The Young’s modulus and Poisson’s ratio of graphene in the armchair and zigzag directions were computed. Table 1 reports the comparison of the Young’s modulus and Poisson’s ratio in armchair and zigzag directions, showing good agreement with values reported in previous studies (900–1050 GPa and 0.14–0.19, respectively).^46,66^ We also investigated the thermal conductivity of the CG graphene with different lengths using reverse nonequilibrium CG-MD simulations. We consider samples with lengths ranging from 10 to 100 nm. The dependence of the inverse thermal conductivity 1/λ to inverse length 1/*L* is illustrated in Figure 6b.

**Table 1:** Mechanical and Thermal Properties of Graphene as Computed by CG-MD

direction	Young’s modulus (GPa)	Poisson ratio (−)	thermal conductivity **λ**_**0**_ (W/(m K))
armchair	845	0.14	621.5
zigzag	916	0.15	658.7

Generally, the relationship between length and thermal conductivity in graphene can be adequately described using the ballistic-to-diffusive crossover formula:^67^
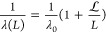
9

Here, *L* is the mean free path of phonon in graphene, and λ_0_ is the thermal conductivity with an infinite length. We make the linear fitting of CG-MD results, as shown in Figure 6b. The simulated value of thermal conductivity at infinite length available in previous studies is 746 W/(m K),^67^ which is only slightly higher than the results from the tested mesoscopic model shown in Table 1. The specific heat capacity of the CG graphene filler was also computed from the enthalpy–temperature plot, as shown in Figure 6c. The best linear fit of this plot had a slope of 434 J/(kg K), whereas the experimentally measured specific heat capacity of graphene was 700 J/(kg K) at temperature of 300 K.^68^ Such discrepancy in the specific heat capacity is due to the reduction in degrees of freedom at the CG level, being an intrinsic limitation of mesoscopic models.^69,70^

**Figure 7:**
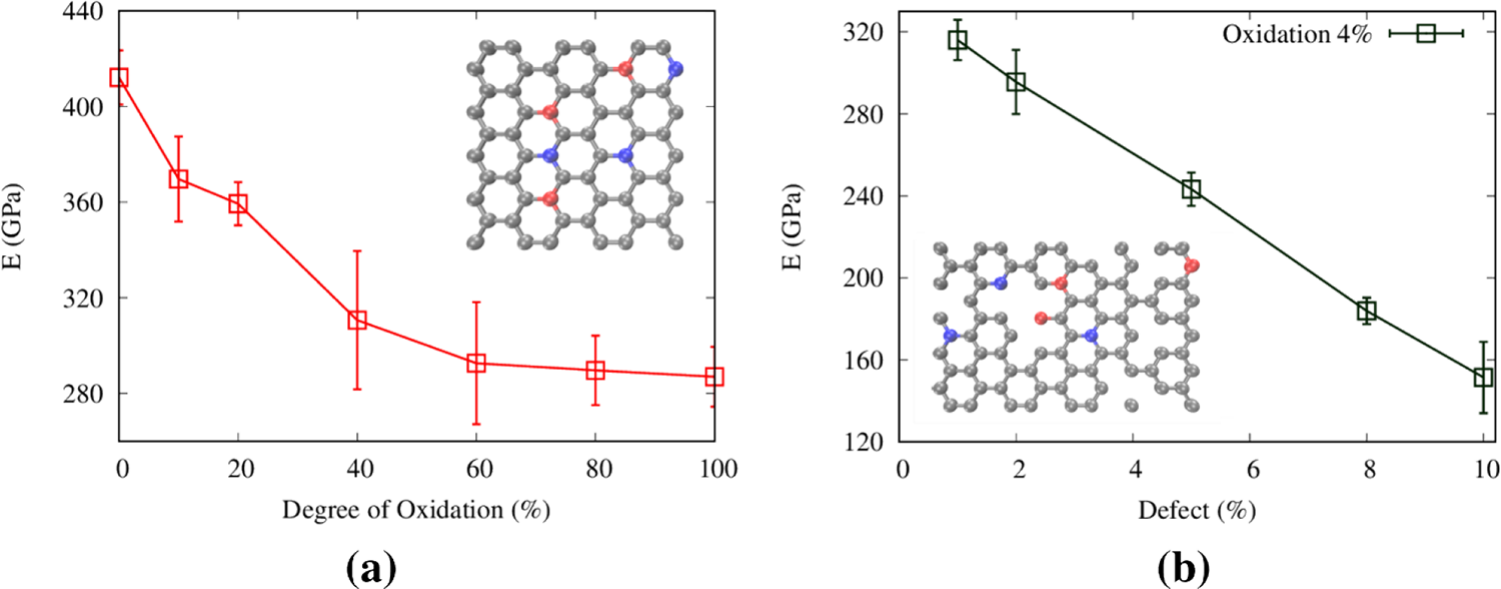
Average values and standard deviation of the (a) Young’s modulus vs degree of oxidation for the CG model of GO and (b) Young’s modulus vs defect percentage for the CG model of rGO. Samples were tested in both armchair and zigzag directions, and results were averaged.

We also evaluated the Young’s moduli of the CG model of graphene oxide and reduced graphene oxide and compared them with the literature evidence. We performed calculations for different degrees of oxidation of GO, and the results show that the Young’s modulus decreases from 412 to 287 GPa with increasing degree of oxidation as shown in Figure 7a, consistent with previous studies.^19^ Note that an equal ratio (1:1) of the hydroxyl-oxidized and epoxide-oxidized beads is considered in the model. Similarly, Figure 7b shows the uniaxial tensile results of rGO with different percentages of defects. We constructed the CG model of rGO by randomly deleting the carbon beads in the CG GO model to generate defects. Note that the monolayer sheet of GO with 4% degree of oxidation was considered for construction of the rGO sheet with different percentages of defects. We observed that the Young’s modulus decreases from 316 to 151 GPa as the percentage of defects in the rGO sheet increases.

### 3.3 Polypropylene Nanocomposites Reinforced by Graphene-Based Fillers

We finally used CG-MD simulation to understand the influence of graphene-based inclusion on the thermal and mechanical properties of pure polypropylene. For PP/Gr, PP/GO, and PP/rGO composites, we randomly introduced PP chains and Gr/GO/rGO sheets into a simulation box, considering different nanofiller concentrations (0.5, 0.8, 1.0, 1.5, and 2.0 wt %). The CG system was then energy minimized through NVT and NPT runs at a temperature of 300 K and pressure of 1 atm (time step of 1 fs; simulation time of 10 ns). The relaxed system was eventually used to determine the thermal and mechanical properties.

**Figure 8:**
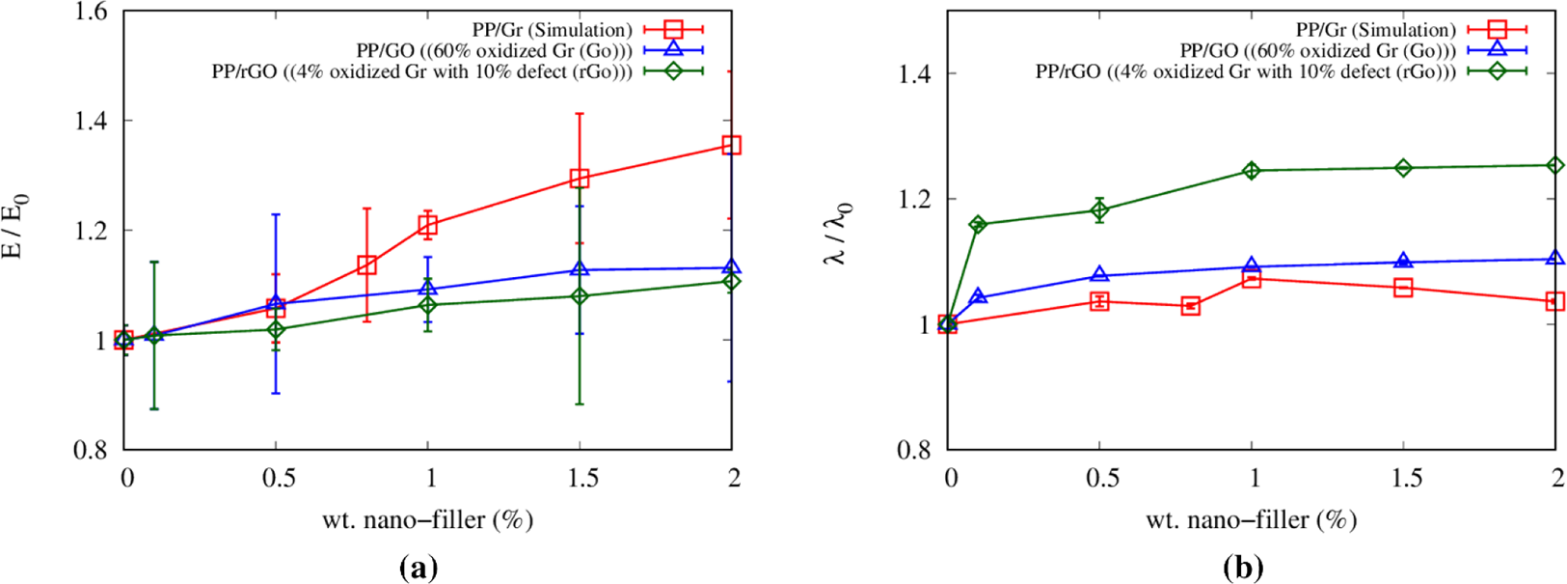
Average values and standard deviation of the relative (a) Young’s modulus and (b) thermal conductivity with respect to the values of pristine PP (*E*_0_ = 0.989 GPa and λ_0_ = 0.138 W m^–1^K^–1^), respectively. Each sample was tested in the *x*, *y*, and *z* directions, and the results were averaged.

Figure 8a shows the overall comparison of relative Young’s moduli of PP/Gr, PP/GO, and PP/rGO nanocomposites. In the case of PP/Gr nanocomposites, the Young’s modulus increases from 5.7% (0.5 wt %) to 35.4% (2.0 wt %), whereas it increases from 3.3% (0.5 wt %) to 13.14% (2.0 wt %) and from 0.8% (0.5 wt %) to 10.71% (2.0 wt %) for PP/GO and PP/rGO, respectively, compared to the pure value of PP. Overall, PP/Gr exhibits better mechanical properties than PP/GO and PP/rGO. In fact, the presence of functional groups in GO deteriorates the mechanical properties, as also observed in a previous study.^19^

Figure 8b shows the comparison of the relative thermal conductivities of PP/Gr, PP/GO, and PP/rGO nanocomposites. Thermal conductivity increases as the weight percentage of nanofillers increases. However, PP/GO and PP/rGO exhibit higher thermal conductivity than PP/Gr. The incorporation of GO into the polymer matrix could significantly improve the original thermal and electrical properties owing to the presence of functional groups leading to enhanced filler–matrix affinity, as also reported by Meng et al.^19^ A detailed list of all CG-MD results is available in Tables S6 and S7.

### 3.4 Comparison and Calibration of Continuum Approaches with Mesoscopic Results

The finite element method is widely used to determine the thermal and mechanical properties of nanocomposites at the macroscopic (continuum) level. FE can be used to calculate the thermal conductivity of nanocomposites by exploiting the Fourier’s law for conduction.^29^ It can also be employed to numerically evaluate the macroscopic mechanical properties of nanocomposites, such as the Young’s modulus and Poisson’s ratio.^29^ For instance, Moghaddam et al.^30^ studied composites with randomly distributed fillers (glass particles) in a polymer matrix (epoxy) by stochastic finite element analysis, exploring the Young’s modulus, Poisson’s ratio, coefficient of thermal expansion, and thermal conductivity. Peng et al.^71^ proposed a numerical-analytical model for the nanoreinforced polymer composites and examined the microstructures and mechanical properties of the composites. Saber-Samandari and Afaghi-Khatibi^72^ used a finite element model to determine the elastic modulus of the nanocomposites with different inclusion shapes, such as platelet, spherical, and cylindrical ones. However, traditional continuum approaches cannot explicitly model the filler–matrix interactions, therefore being unable to represent the effect of different physical–chemical features of the interface. Mesoscopic simulations allow overcoming this issue because they model the filler–matrix interaction with molecular precision. Here, for the sake of completeness, we first compare the predictions from our CG model against those from traditional continuum predictions.

**Figure 9:**
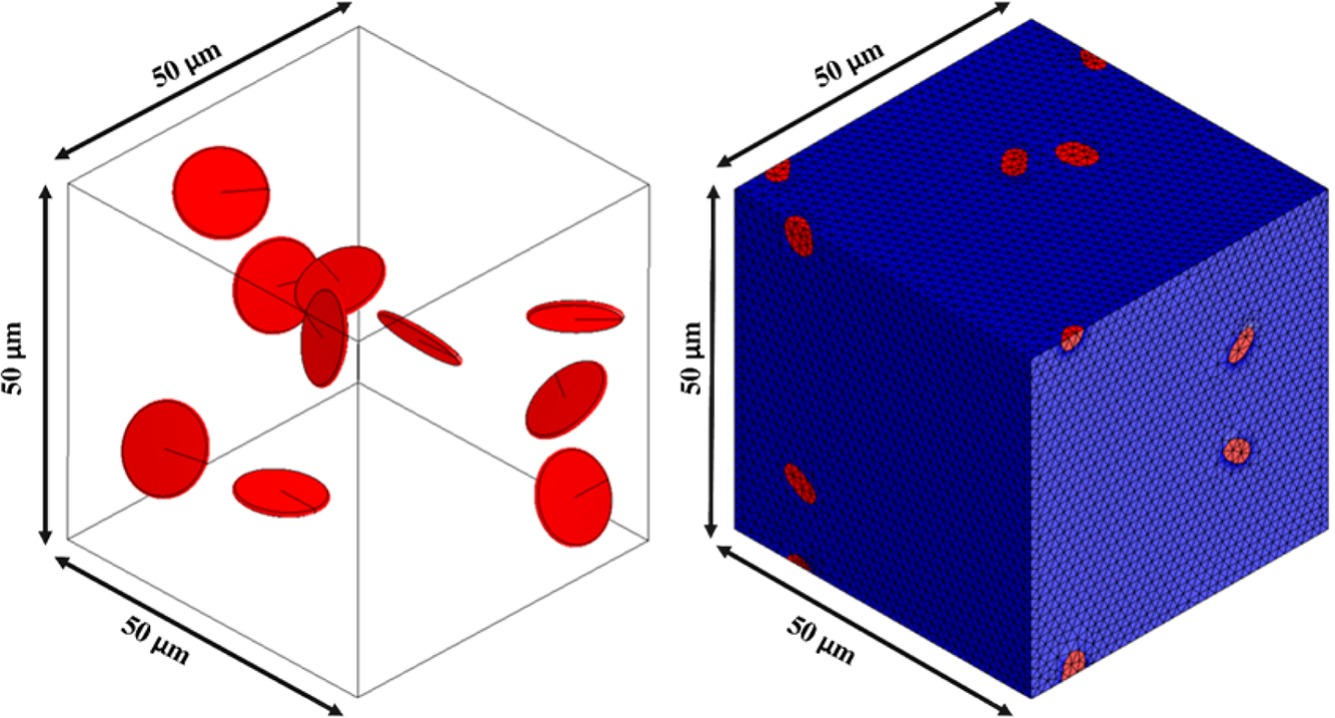
Example of computational domain and resulting mesh for the finite element model of polypropylene (blue) reinforced by graphene (red).

Hence, we used continuum models to evaluate the Young’s modulus, Poisson’s ratio, and thermal conductivity for the considered PP/Gr nanocomposites. The input parameters were taken coherently with CG models as follows: polypropylene: density 1.15 g/cm^3^, Young’s modulus 0.99 GPa, Poisson’s ratio 0.43, and thermal conductivity 0.13 W/(m K); graphene: density 1.40 g/cm^3^, aspect ratio 10, Young’s modulus 916 GPa, Poisson’s ratio 0.15, and thermal conductivity 8.75 W/(m K). The RVE model was then generated similarly to the CG configurations by choosing the mass fraction (0.5, 0.8, 1.0, 1.5) wt % and thus the number of graphene inclusions dispersed randomly in the PP matrix (see Figure 9).

**Figure 10:**
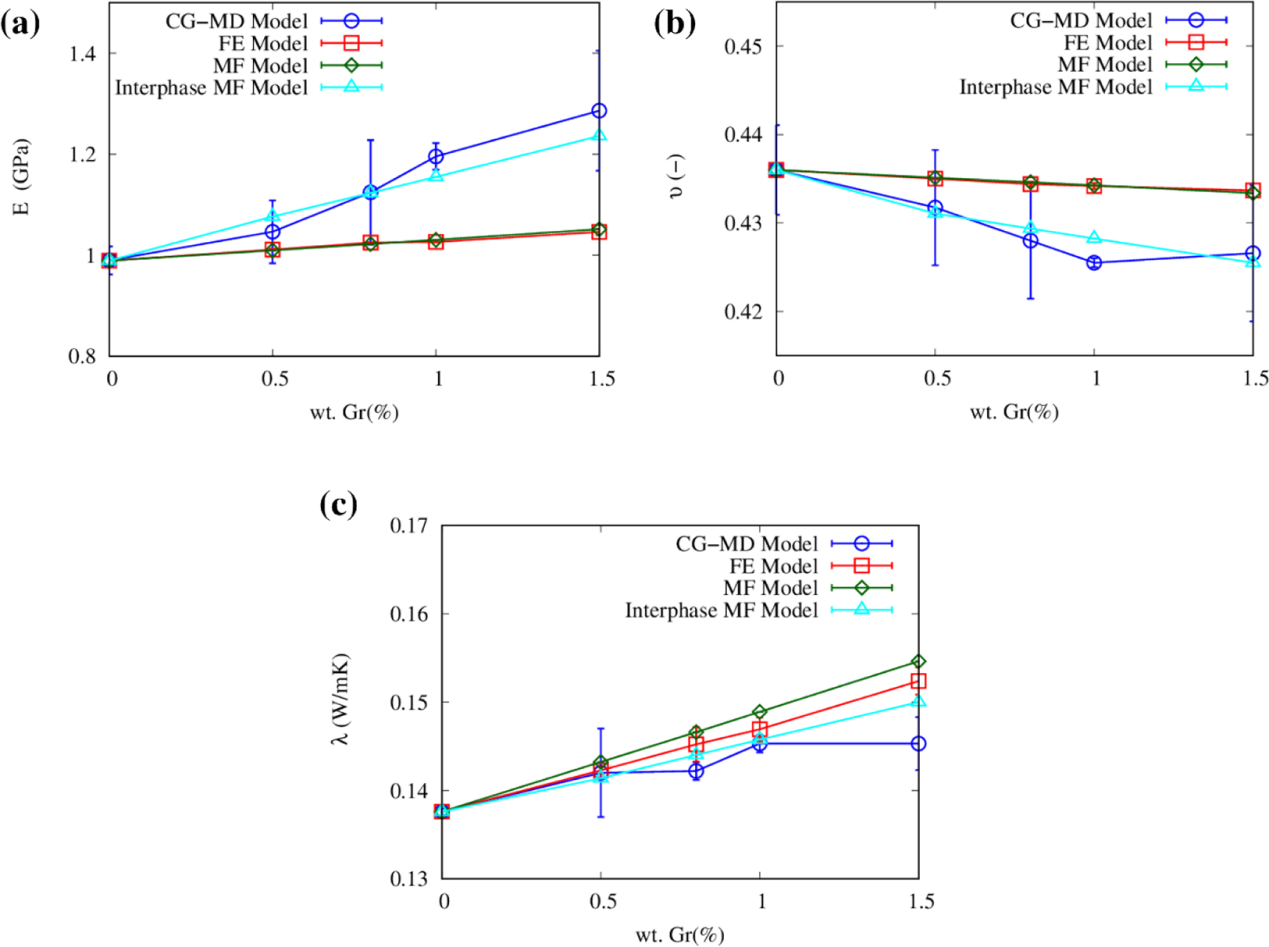
Average values with standard deviation of the relative (a) Young’s modulus, (b) Poisson’s ratio, and (c) thermal conductivity enhancement of PP reinforced with graphene. Coarse-grained (CG), finite element (FE), and mean field (MF) results (considering either filler and matrix phases, or filler, matrix, and interphase) are compared. Each sample was tested in *x*, *y*, and *z* directions, and results were averaged (see also Figure S4).

The results obtained from the FE and MF models are compared to the CG simulations in Figure 10. The Young’s modulus increases from 5.7% (0.5 wt %) to 35.4% (2.0 wt %) compared with the neat PP in CG-MD simulation, whereas it increases from 2.2% (0.5 wt %) to 8.0% (2.0 wt %) with the FE and MF models (see Figure 10a). Hence, both FE and MF models underestimate the Young’s modulus enhancement provided by graphene inclusions, whereas the proposed CG model takes into consideration the filler–matrix interaction with molecular precision. The Poisson’s ratio of PP/Gr composites, as predicted by the mesoscopic and continuum models, is shown in Figure 10b. The CG, FE, and MF models all predict a progressive reduction of Poisson’s ratio with increasing graphene concentrations, with the CG simulation predicting higher reduction compared to both FE and MF models.

To consider the effect of matrix–filler interactions also in continuum approaches, Ji et al.^73^ proposed an interphase micromechanical model based on the Takayanagi^74−76^ homogenization technique while accounting for interfacial contribution. Following the Ji et al. approach, we have introduced an interphase in the Mori–Tanaka MF model, considering a thickness of 0.5 nm from the fiber surface as observed from the radial distribution function of PP around the fillers in our CG-MD simulations. Numerous studies^77−80^ have revealed that the polymeric region in the proximity of the filler (i.e., interphase) exhibits significantly enhanced elastic modulus when transitioning from the nanoparticles to the polymer matrix. It is crucial to consider such interfacial stiffening, as neglecting it could lead to inaccurate predictions of the overall nanocomposite properties. To this purpose, the CG-MD results were taken as a reference to best fit the mechanical properties of the interphase included in the MF model, which resulted in a Young’s modulus of 5 GPa and a Poisson’s ratio of 0.44, clearly showing enhanced mechanical properties of the interphase. Results in Figures 10a,b demonstrate a good agreement between the CG-MD and the calibrated interphase MF model, which is finally capable to accurately reproduce the effect of filler–matrix interface on the mechanical properties of the nanocomposite.

Figure 10c compares the thermal conductivity of PP/Gr composites predicted by the mesoscopic and continuum approaches. The thermal conductivity of the PP composite material increases with the weight percentage of graphene fillers in all cases, with CG-MD predictions showing lower enhancement because thermal boundary resistances at the filler–filler and filler–matrix interfaces are duly considered in this model while being neglected by continuum ones.^81,82^

To include the effect of thermal boundary resistance also in continuum approaches, Shahil and Balandin^83^ proposed a modified Maxwell–Garnett Effective Medium Approximation (MG-EMA) incorporating an interphase with thermal properties dictated by the Kapitza resistance at the interface between epoxy and graphene, also considering the effect of size and aspect ratio of fillers (see eq 1 in ref (83) for details). In the realm of predicting effective properties of composite materials, including the effective thermal conductivity, MG-EMA stands out as a widely employed approach. This theoretical framework is relevant when a host material (matrix) accommodates dispersed inclusions (fillers) composed of different materials. It is worth noting that the MG-EMA concept can be traced back to the classical models developed by Maxwell and Lord Rayleigh. However, the modern incarnation of MG-EMA considers additional factors such as interphase thermal resistance, filler topologies, and orientations within the matrix. This updated framework accounts for various arbitrarily shaped fillers, offering a more comprehensive and accurate representation of heat conduction in composite materials.^84−86^

Following the methodology outlined by Shahil and Balandin, we incorporated an interphase in the Mori–Tanaka MF model, adopting a thickness consistent with the mechanical model (i.e., 0.5 nm). Then, the Kapitza resistance of the interphase between polymer and graphene was taken as 3.7 × 10^–9^ m^2^K/W, consistent with previous observations in the literature.^83^ This led to a thermal conductivity of the interface of about 0.135 W/(m K), which is similar to the CG thermal conductivity of neat PP. The inclusion of thermal boundary resistance at the filler–matrix interface improves the agreement between the interphase MF model and the CG simulation result. A detailed list of all such model comparisons is available in Tables S7–S9.

### 3.5 Multiscale Model Validation

We also compared the Young’s modulus and thermal conductivity predicted by the calibrated Mori–Tanaka interphase MF model with the experimental results of the tested PP/GNP nanocomposites. The model input parameters were taken consistently with experimental data, with polypropylene having a density 0.900 g/cm^3^, Young’s modulus 0.944 GPa, Poisson’s ratio 0.45, and thermal conductivity 0.23 W/(m K). Graphene nanoplatelets (GNPs) were characterized by a density of 2.2 g/cm^3^, Young’s modulus 1,030 GPa, Poisson’s ratio 0.19, and thermal conductivity 3,000 W/(m K). The interphase properties were taken from the calibration carried out in Section 3.4, leveraging the outcomes of the mesoscopic model.

It is important to note that the aspect ratio of GNPs often deviates significantly from its nominal value upon incorporation into the polymer matrix. For instance, in a study by Kalaitzidou et al.^87^ investigating the elastic modulus of xGnP-15/PP composites experimentally, the predicted values from both the Halpin–Tsai and Tandon–Weng models overestimated the experimental results. This discrepancy was attributed to the use of the nominal aspect ratio (∼1500) of xGnP-15 in the calculations, whereas the effective aspect ratio was found to be at least 1 order of magnitude smaller due to filler aggregation during composite processing. The nominal aspect ratio refers to the aspect ratio of the reinforcing particles as they are manufactured, whereas the effective aspect ratio takes into account the actual configuration of the reinforcing particles within the composite material. The effective aspect ratio is more relevant when assessing how a composite material performs in practical applications. Similarly, Jun et al.^88^ examined thermal, mechanical, and electrical properties of PP/GNP composites containing large-sized GNPs (aspect ratio ∼7500) via melt compounding. Here, the predicted modulus of the composites using the Halpin–Tsai model exceeded the experimental values because of the significantly reduced aspect ratio of GNPs within the composites. However, by employing the aspect ratio measured after composite processing (∼50) rather than the nominal aspect ratio, a substantial improvement in agreement between predicted and experimental values was achieved. In another investigation by Innes et al.^89^ focusing on a rubber matrix reinforced with two types of GNPs (M5 and M15) having lateral dimensions of 5 and 15 μm, with an average thickness in the range of 6–8 nm, micromechanical modeling revealed a relationship between GNP aspect ratio and mechanical properties. The effective aspect ratio contributing to the enhancement of the elastic modulus was determined to be 79 and 86 for M5 and M15 GNPs, respectively, which were significantly smaller than the nominal values.

**Figure 11:**
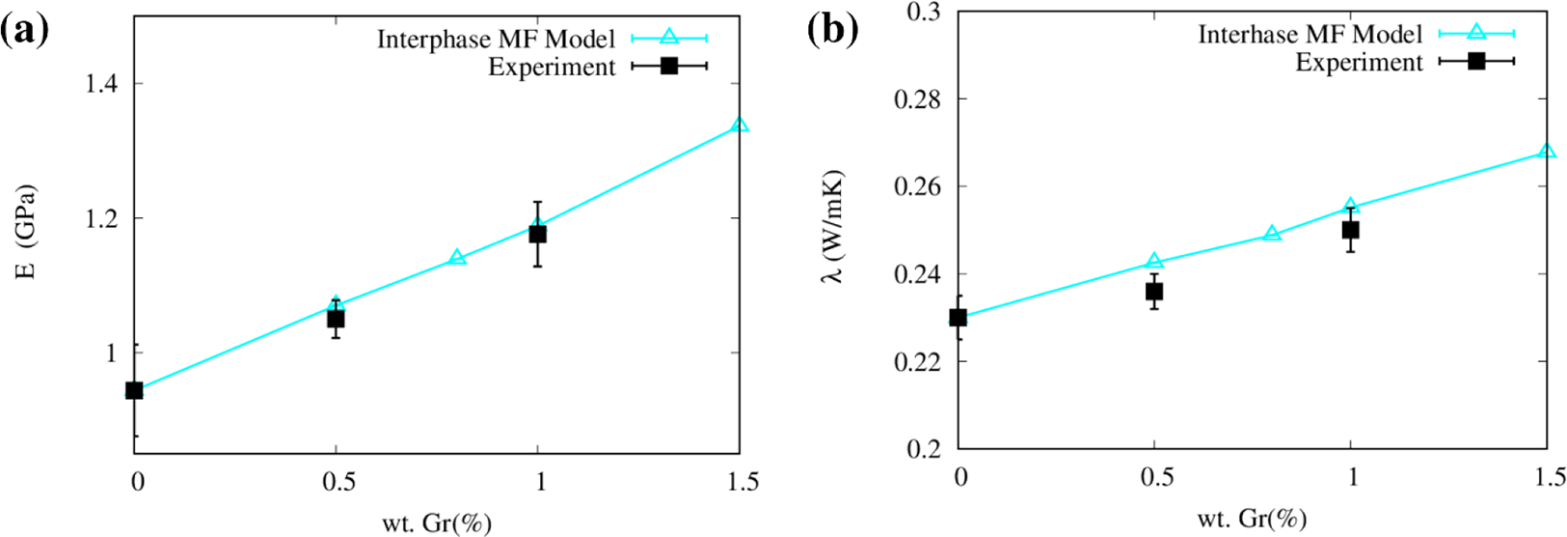
Average values with standard deviation of the (a) Young’s modulus and (c) thermal conductivity enhancement of PP reinforced with GNPs. Results from experimental measures and interphase MF model predictions are compared (tabulated results are reported in Tables S10 and S11).

These findings highlight the importance of adjusting the nominal aspect ratio of GNP fillers after their inclusion in the polymer matrix. Such adjustments are crucial for achieving accurate predictions of the properties in the resulting composites. In this study, the Mori–Tanaka interphase MF model showed the best prediction accuracy of elastic modulus and thermal conductivity of experimental PP/GNP samples (cf. Figure 11) when an effective aspect ratio of 50 was considered, in line with previous studies.^87^

## 4 Conclusions

In this study, we introduce and validate an innovative mesoscopic model for polypropylene nanocomposites reinforced with graphene derivatives, enabling the prediction of their thermal and mechanical properties. The CG-MD simulation method was employed to explore the material properties of both the graphene-based nanofillers and pure polypropylene at the mesoscopic level with the goal of predicting the properties of the resulting nanocomposite materials.

The mesoscopic simulations revealed that the developed CG model of PP accurately reproduces the glass transition temperature, Young’s modulus, Poisson’s ratio, and thermal conductivity, exhibiting significant accuracy except for an overestimation in density. Additionally, we computed the mechanical and thermal properties of graphene by using CG potentials, and our results align well with values reported in the literature. Furthermore, we extended our analysis to encompass graphene oxide and reduced graphene oxide by employing the same CG force-field. This enabled us to predict the degradation in mechanical behavior as the degree of oxidation in graphene-based fillers increases.

The CG model of both polypropylene- and graphene-based nanofillers was employed to compute the thermal and mechanical properties of graphene-reinforced polypropylene composites. The CG model predicts that an increase in the nanofiller reinforcement percentage in polypropylene leads to an improved mechanical and thermal behavior of the resulting nanocomposite. Our study highlights that the graphene-reinforced polypropylene (PP/Gr) composite exhibits more optimized mechanical properties compared to the composites reinforced with graphene oxide (PP/GO) or reduced graphene oxide (PP/rGO) at similar weight percentages of reinforcement. Conversely, the PP/rGO composite demonstrates superior thermal behavior compared with PP/GO and PP/Gr nanocomposites. To validate the CG predictions and improve prediction accuracy beyond traditional continuum approaches, we utilized the MF model with interphase. This model incorporates calibrated properties based on the results obtained from the mesoscopic model, enabling a detailed representation of the filler–matrix interactions. As a result, the MF model with interphase demonstrated an enhanced prediction capability, and its outcomes were validated against experimental data. This validation process corroborated the accuracy of our findings for predicting the thermal and mechanical properties of graphene-reinforced polypropylene nanocomposites.

Our work sets the stage for further studies on the multiscale modeling of thermal and mechanical properties of PP nanocomposites reinforced by a broader combination of nanofillers, e.g., carbon nanotubes. Interestingly, our work can have an impact in the energy field. Particularly, additivation of phase-change materials remains an open issue for unlocking the full potential of latent and other heat storage applications.^90,91^

## Data Availability

All data generated or analyzed during this study are included in this published article and in the Supporting Information file. All simulation files and postprocessing codes are available at the Zenodo archive associated with this work (DOI: 10.5281/zenodo.7327415).

## Supporting Information Available

Coarse-grained potentials and tabulated results (PDF)

## Notes

The preprint of this work is available on arXiv (DOI: 10.48550/arXiv.2211.13148).
