# Supplementary material for: Mesoscopic Modeling and Experimental Validation of Thermal and Mechanical Properties of Polypropylene Nanocomposites Reinforced By Graphene-Based Fillers
Source: Macromolecules. 2023 Dec 6;56(24):9969–82. doi: 10.1021/acs.macromol.3c01529 (PMC10753874; doi:10.1021/acs.macromol.3c01529)
Supplement: Supplementary file 1 — ma3c01529_si_001.pdf [file ma3c01529_si_001.pdf]

# - Supplementary Information -

Mesoscopic modeling and experimental validation of thermal and mechanical properties of polypropylene nanocomposites reinforced by graphene-based fillers

Atta Muhammad[a,b], Rajat Srivastava[a,c], Nikolaos Koutroumanis[d], Dionisis Semitekolos[e], Eliodoro Chiavazzo[a], Panagiotis-Nektarios Pappas[d], Costas Galiotis[d,f], Pietro Asinari[a,g], Costas A. Charitidis[e], Matteo Fasano[a*]

[a] *Department of Energy, Politecnico di Torino, Corso Duca degli Abruzzi 24, 10129, Torino, Italy.*

[b] *Department of Mechanical Engineering, Mehran University of Engineering and Technology, SZAB Campus, 66020 Khairpur Mir's, Sindh, Pakistan.*

[c] *Department of Engineering for Innovation, University of Salento, Piazza Tancredi 7, 73100, Lecce, Italy.*

[d] *Institute of Chemical Engineering Sciences, Foundation of Research and Technology-Hellas, Stadiou str. Rion, 26504, Patras, Greece*

[e] *School of Chemical Engineering, National Technical University of Athens, 9 Heroon Polytechniou, 15780 Athens, Greece*

[f] *Department of Chemical Engineering, University of Patras, 1 Caratheodory, 26504 Patras, Greece*

[g] *Istituto Nazionale di Ricerca Metrologica, Strada delle Cacce 91, 10135 Torino, Italy.*

[*] Corresponding author, email address: matteo.fasano@polito.it

SUPPLEMENTARY NOTE 1: COARSE-GRAINED POTENTIALS

i. Polypropylene

k_b [kcal/mol Å^2]	r_0 [Å]	k_θ [kcal/(mol rad^2)]	θ_0 [°]	k_ϕ, ϕ_s, n [kcal/mol][°] [−]
57.36	2.98	9.32	119	0.74, 100, 1; -1.41, 190, 2

Table S1. Parameters of bonded interactions in the PP model.[1]

σ [Å]	ε [kcal/mol]
4.3	0.625

Table S2. Parameters of non-bonded interactions in the PP model.[1]

ii. Graphene

Interactions	Parameters
Bond	$d_0 = 2.8$ Å<br>$D_0 = 196.38$ kcal/mol<br>$\alpha = 1.55$ Å^{-1}<br>$d_{cut} = 3.25$ Å
Angle	$\theta_0 = 120°$<br>$k_\theta = 409.40$ kcal/(mol rad^2)
Dihedral	$k_\emptyset = 4.15$ kcal/mol; $\phi_s = 0°$; $n = 2$

Table S3. Parameters of bonded interactions in the graphene model, where d_{cut} is the bond cutoff (failure criterion) that corresponds to the maximum force on the bond.[2]

Interactions	Parameters
Non-bonded	$\sigma_{lj} = 3.46$ Å<br>$\varepsilon_{lj} = 0.82$ kcal/mol<br>$r_{cut} = 12$ Å

Table S4. Parameters of non-bonded interactions in the graphene model, where r_{cut} is the cutoff radius.[2]

iii. Graphene oxide and reduced graphene oxide

<table>
<tr><th>Interaction</th><th>Functional Form</th></tr>
<tr><td>Bond</td><td>Type I potential and parameters:

$$V_{b.I}(d) = D_0[1 - e^{-\alpha(d-d_0)}]^2$$

$$d_0 = 2.86 \text{ Å}$$

$$D_0 = 443.07 \text{ kcal/mol}$$

$$\alpha = 1.54 \text{ Å}^{-1}$$

$$d_{cut} = 3.7 \text{ Å}$$

Type II & III:

$$V_{b,II\&III}(d) = \begin{cases} k_{be}(d-d_0)^2;\ d < d_{c1} \\ k_{bp}(d-d_{c1})^2 + 2k_{be}(d_{c1}-d_0)(d-d_{c1}) + C_1;\ d_{c2} < d < d_{c2} \\ k_{bf}(d-d_{c2})^2 + \left[2k_{bp}(d_{c2}-d_{c1}) + 2k_{be}(d_{c1}-d_0)\right](d-d_{c2}) + C_2;\ d > d_{c2} \\ C_1 = k_{be}(d_{c1}-d_0)^2 \\ C_2 = k_{bp}(d_{c2}-d_{c1})^2 + 2k_{be}(d_{c1}-d_0)(d_{c2}-d_1) + C_1 \end{cases}$$

Type II parameters:

$$d_0 = 2.94 \text{ Å}$$

$$d_{c1} = 3.12 \text{ Å}$$

$$d_{c2} = 3.46 \text{ Å}$$
</td></tr>
</table>

	$k_{be} = 317.34\ \text{kcal/mol}\ \mathring{A}^2$<br>$k_{bp} = 126.94\ \text{kcal/mol}\ \mathring{A}^2$<br>$k_{bf} = 634.68\ \text{kcal/mol}\ \mathring{A}^2$<br>Type III parameters:<br>$d_0 = 2.80\ \mathring{A}$<br>$d_{c1} = 3.00\ \mathring{A}$<br>$d_{c2} = 4.20\ \mathring{A}$<br>$d_{cut} = 4.3\ \mathring{A}$<br>$k_{be} = 256.10\ \text{kcal/mol}\ \mathring{A}^2$<br>$k_{bp} = 21.34\ \text{kcal/mol}\ \mathring{A}^2$<br>$k_{bf} = 512.20\ \text{kcal/mol}\ \mathring{A}^2$
Angle	$V_a(\theta) = k_\theta(\theta - \theta_0)^2$<br>$\theta_0 = 120^0$<br>Type I parameter: $k_\theta = 456.61 \frac{\text{kcal}}{\text{mol}}$<br>Type II parameter: $k_\theta = 259.47 \frac{\text{kcal}}{\text{mol}}$<br>Type III parameter: $k_\theta = 189.93 \frac{\text{kcal}}{\text{mol}}$
Non-bonded	$V_{nb} = 4\varepsilon_{lj}\left[\left(\frac{\sigma_{lj}}{r}\right)^{12} - \left(\frac{\sigma_{lj}}{r}\right)^{6}\right]$<br>$\sigma_{lj} = 7.48\ \mathring{A}$<br>Type C parameter: $\varepsilon_{lj} = 0.0255\ \text{kcal/mol}$<br>Type H parameter: $\varepsilon_{lj} = 0.128\ \text{kcal/mol}$<br>Type E parameter: $\varepsilon_{lj} = 0.0797\ \text{kcal/mol}$

Table S5. Functional forms and parameters of coarse-grained model of graphene oxide and reduced graphene oxide.[3]

SUPPLEMENTARY NOTE 2: GRAPHENE NANOPLATELETS

Graphene was used as a nanofiller in the polymer matrix. The graphene nanoplatelets (GNPs) production was carried out by using a commercially available shear laboratory mixer by Silverson. The raw material for the production process is graphite powder which was obtained from NGS Naturgraphit GmbH, Germany, with a particle average lateral size of 500 microns.

The main component of the mixing apparatus is a 4-blade rotor placed within a fixed screen (stator), which aims to apply the required shear stress field for the graphite exfoliation to take place. After the graphite powder is weighed into the mixing vessel, the required aqueous-based exfoliating liquid containing surfactant (Triton-X) is added. At the initial stage of the shear exfoliation process, the mixer head is driven towards the liquid solution into the vessel and operates in low rotational speed. The speed is gradually increased from 1000 rpm to 5000 rpm and the system runs at high speed for about 40 minutes. Finally, the mass of the produced exfoliated graphene is measured after drying at 80 °C for 24 h under vacuum conditions.

The carbon content of produced graphene platelets is about 91%. The GNPs powder dispersed on the adhesive conductive tape for scanning electron microscope (SEM) characterization is shown in Figure S1: the platelets have a lateral dimension within the range of 2-5 μm, and a thickness of 5-7 nm (see Figure S2). The material appears to be partially exfoliated (or reaggregated) with platelets showing highly exfoliated regions together with thicker crystals.

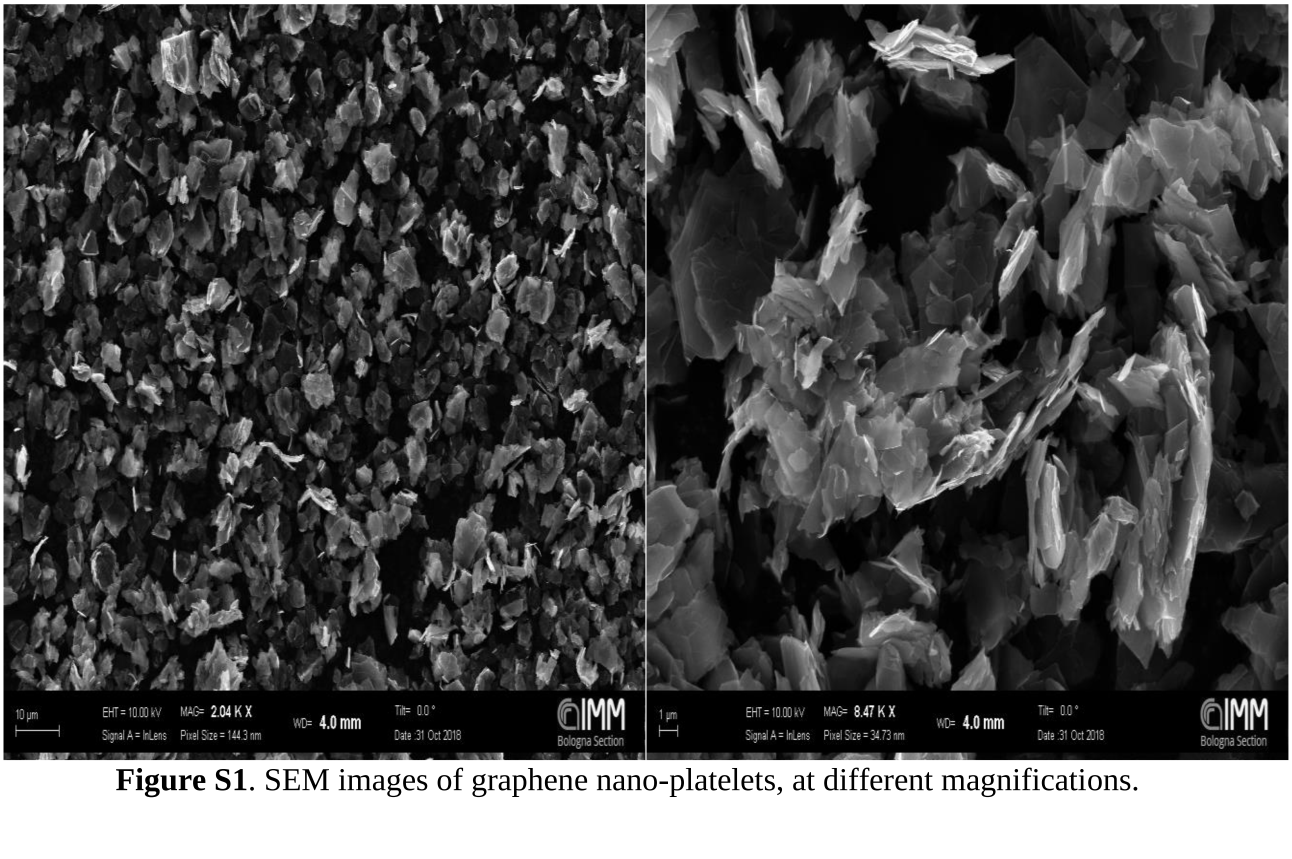

Figure S1. SEM images of graphene nano-platelets, at different magnifications.

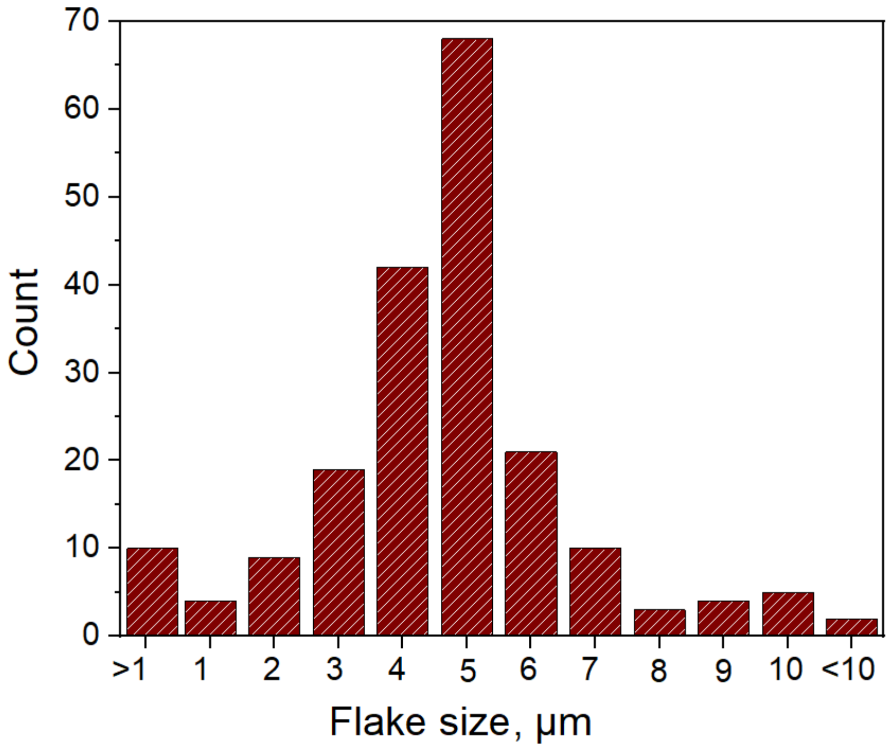

Figure S2. Size distribution of the graphene platelets.

SUPPLEMENTARY NOTE 3: EXPERIMENTAL VS. MODELLING RESULTS

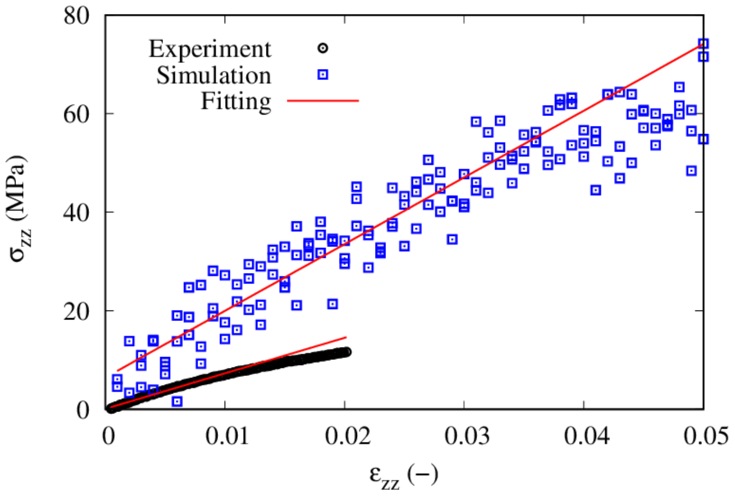

Figure S3. Stress vs. strain curve in the elastic region for the pristine PP.

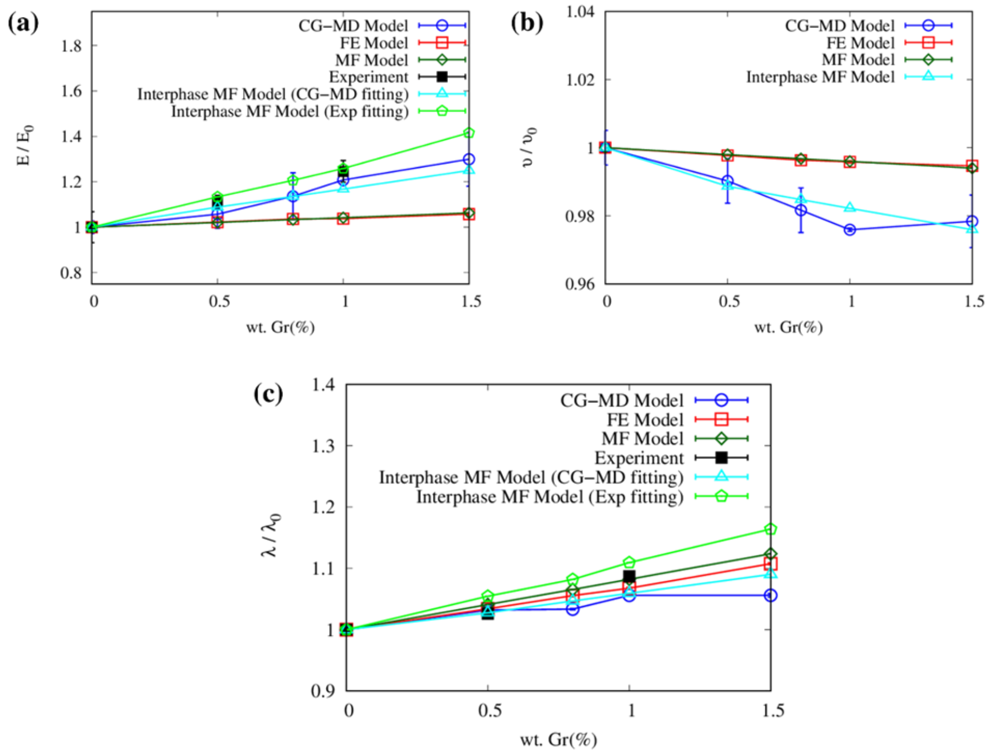

Figure S4. Average values with standard deviation of relative (a) Young's modulus, (b) Poisson's ratio, and (c) thermal conductivity enhancement of PP reinforced with graphene with respect to the values of pristine PP. Coarse-Grained (CG), Finite Element (FE), Mean Field (MF) and experimental results are compared.

SUPPLEMENTARY NOTE 4: TABULATED RESULTS

**wt.%**	**Young's modulus, E (GPa)**		**Thermal conductivity, λ (W/mK)**	
	**PP/GO**	**PP/rGO**	**PP/GO**	**PP/rGO**
**0.0**	0.989 (0.027)	0.989 (0.027)	0.138 (0.003)	0.138 (0.003)
**0.1**	1.022 (0.030)	0.997 (0.134)	0.143 (0.005)	0.160 (0.004)
**0.5**	1.054 (0.163)	1.008 (0.038)	0.148 (0.001)	0.163 (0.019)
**1.0**	1.080 (0.059)	1.052 (0.048)	0.150 (0.001)	0.171 (0.009)
**1.5**	1.115 (0.116)	1.068 (0.197)	0.151 (0.003)	0.172 (0.001)
**2.0**	1.119 (0.207)	1.095 (0.021)	0.152 (0.001)	0.173 (0.001)

Table S6. CG-MD results of PP/GO and PP/rGO nanocomposites.

**wt.%**	**CG MD E (GPa)**	**Continuum (MF) E (GPa)**	**Continuum (FE) E (GPa)**	**Continuum (Interphase MF) E (GPa)**
**0.0**	0.989 (0.027)	0.989	0.989(0.027)	0.989
**0.5**	1.046 (0.062)	1.0096	1.011(0.001)	1.076
**0.8**	1.124 (0.103)	1.022	1.024(0.001)	1.123
**1.0**	1.196 (0.026)	1.0303	1.026(0.000)	1.154
**1.5**	1.28 (0.118)	1.0513	1.045(0.006)	1.235
**2.0**	1.34 (0.134)	1.0724	1.069(0.005)	1.319

Table S7. Comparison of Young's modulus obtained from coarse-grained and continuum simulations of PP/Gr.

wt.%	CG MD<br><br>Poisson's ratio	Continuum (MF) Poisson's ratio	Continuum (FE) Poisson's ratio	Continuum (Interphase MF) Poisson's ratio
**0.0**	0.436 (0.005)	0.436	0.436 (0.005)	0.436
**0.5**	0.431 (0.006)	0.435	0.435 (0.000)	0.431
**0.8**	0.428 (0.006)	0.434	0.434 (0.000)	0.429
**1.0**	0.425 (0.000)	0.434	0.434 (0.000)	0.428
**1.5**	0.426 (0.007)	0.433	0.433 (0.000)	0.425
**2.0**	0.427 (0.004)	0.432	0.432 (0.000)	0.422

Table S8. Comparison of Poisson's ratio obtained from coarse-grained and continuum simulations of PP/Gr.

wt.%	CG MD<br><br>λ (W/mK)	Continuum (MF)<br><br>λ (W/mK)	Continuum (FE)<br><br>λ (W/mK)	Continuum (Interphase MF)<br><br>λ (W/mK)
**0.0**	0.137 (0.000)	0.137	0.137 (0.000)	0.137
**0.5**	0.142 (0.008)	0.143	0.142 (0.000)	0.141
**0.8**	0.142 (0.000)	0.146	0.145 (0.001)	0.144
**1.0**	0.145 (0.002)	0.148	0.146 (0.001)	0.145
**1.5**	0.145 (0.000)	0.155	0.152 (0.001)	0.15
**2.0**	0.144 (0.003)	0.160	0.155 (0.002)	0.154

Table S9. Comparison of thermal conductivity obtained from coarse-grained and continuum simulations PP/Gr.

wt.%	Continuum (Interphase MF)<br>E (GPa)	Experimental<br>E (GPA)
**0.0**	0.944	0.944 (0.068)
**0.5**	0.107	1.05 (0.028)
**0.8**	1.139	-
**1.0**	1.188	1.176 (0.048)
**1.5**	1.336	-

Table S10. Comparison of Young's modulus obtained from experiments and continuum simulations of PP/GNPs.

wt.%	Continuum (Interphase MF)<br>λ (W/mK)	Experimental<br>λ (W/mK)
**0.0**	0.23	0.23 (0.005)
**0.5**	0.242	0.236 (0.003)
**0.8**	0.248	-
**1.0**	0.255	0.250 (0.005)
**1.5**	0.267	-

Table S11. Comparison of thermal conductivity obtained from experiments and continuum simulations PP/GNPs.

REFERENCES

(1) Panizon, E.; Bochicchio, D.; Monticelli, L.; Rossi, G. MARTINI Coarse-Grained Models of Polyethylene and Polypropylene. *J Phys Chem B* **2015**, *119* (25), 8209–8216.

(2) Ruiz, L.; Xia, W.; Meng, Z.; Keten, S. A Coarse-Grained Model for the Mechanical Behavior of Multi-Layer Graphene. *Carbon N Y* **2015**, *82*, 103–115.

(3) Meng, Z.; Soler-Crespo, R. A.; Xia, W.; Gao, W.; Ruiz, L.; Espinosa, H. D.; Keten, S. A Coarse-Grained Model for the Mechanical Behavior of Graphene Oxide. *Carbon N Y* **2017**, *117*, 476–487.
